# Endocrine-disrupting organochlorine xenobiotics in fish products imported from Asia—an assessment of human health risk

**DOI:** 10.1007/s10661-021-08914-5

**Published:** 2021-02-16

**Authors:** Agata Witczak, Daiki Harada, Aleksandra Aftyka, Jacek Cybulski

**Affiliations:** 1 Department of Toxicology, Dairy Technology and Food Storage, Faculty of Food Sciences and Fisheries, West Pomeranian University of Technology, Szczecin, Poland; 2 Laboratory of Regulation of Metabolism and Behavior, Faculty of Agriculture, Kyushu University, Fukuoka, 819-0395 Japan

**Keywords:** Imported fish products, Endocrine-disrupting compounds, Organochlorine pesticides, Polychlorinated biphenyls, Assessment of human health risk

## Abstract

The sources of endocrine-disrupting persistent organochlorine compounds (OC) are environmental pollutants. Contaminated food is a direct result of environmental pollution, and fish are considered as the main source of OC in the human diet. This study aimed to analyze the contamination of imported fish fillets with organochlorine pesticides (OCPs) and polychlorinated biphenyl (PCB) congeners in the context of potential health risks of consumers in Poland in the light of the new tolerable weekly intake (TWI) values. The tested compounds in fish products were determined by liquid-liquid extraction and gas chromatography mass spectrometry (GS-MS) method. Despite the detection of almost all pesticides analyzed in the fish fillets tested, the risk factor (hazard quotient) was significantly lower than 1.0, ranging from 0.003 to 0.013. Considering the previous recommended TWI value (14 pg-TEQ/kg bw/week), the estimated weekly intake was lower at 43–53% of TWI. However, according to the new TWI values set by the EFSA in 2018, the estimated weekly intake was about three times higher than the TWI. This raises concerns regarding threats to consumer health.

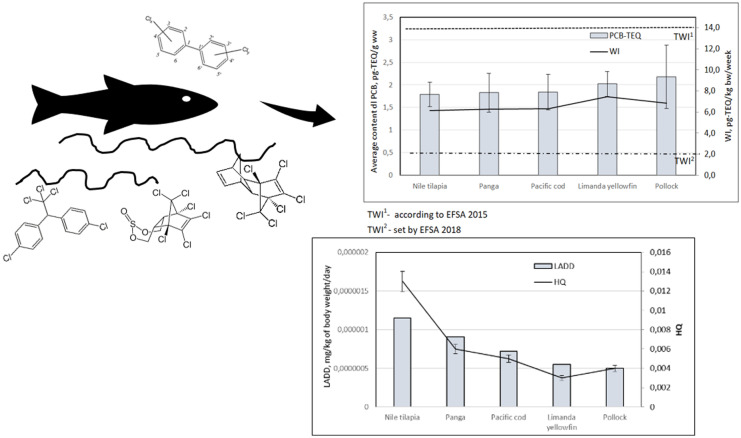

## Introduction

In recent years, persistent organic pollutants (POPs) have been the focus of great concern partly because of the toxic effects induced by organochlorine compounds. The most important were the organochlorine pesticides (OCPs) that were used commonly in agricultural production process and, i.e., mosquito control in the twentieth century. Polychlorinated biphenyls (PCBs) were applied widely in many industries, particularly in electrotechnics (EFSA, 2005; Faroon & Ruiz, 2016). The characteristics of these compounds are similar regarding their extreme persistence, good solubility in fat, and high bioaccumulation in living organisms (Barr et al., 2007; Salem et al., 2009; Lagalante et al., 2009; Walczak & Reichert, 2016). Both groups of these compounds were considered persistent organic pollutants (POPs) (Stockholm Convention, 2018).

These compounds have been shown to exhibit mainly estrogenic and mutagenic or partly carcinogenic properties in animals (Gregoraszczuk et al., 2003). Organochlorine xenobiotic residues are often detected in foods, and especially in fish products (Deribe et al., 2014; Cui et al., 2015). Due to the specific physico-chemical properties of the organochlorine compounds (OC), the frequent consumption of contaminated fish can increase the health risk of consumers.

Fish and seafood are considered healthier alternatives to meat and meat products. According to FAO, in 2010–2015, the global production of fish and other aquatic organisms increased 15% from 147 to 168.7 million tonnes (World aquaculture, 2010; FAO, 2018). The OECD–FAO estimated that, in 2017–2026, fish consumption in the EU will increase by about 5% thanks to the ongoing economic recovery and changes in the nutrition trends of Europeans (OECD/FAO, 2017).

Based on the chemical structure of OCPs, the main groups include chloroethylene hydrocarbons; dichlorodiphenyls—DDT, and methoxychlor; cyclodienic derivatives—aldrin, dieldrin, heptachlor, endosulfan, and chlordan; cycloparaffinic derivatives—HCH and lindane; and chlorinated terpenes—toxaphene. The presence of harmful compounds –particularly the most durable metabolite of DDT (p,p’DDE), the hexachlorocyclohexane isomer (β-HCH), and HCB is evident in all environmental samples and in materials derived from human beings worldwide.

On the other hand, there are 209 individual congeners of PCBs, which include non-dioxin-like PCBs (ndl-PCBs) and dioxin-like PCBs (dl-PCBs including mono-ortho and non-ortho congeners). These compounds differ in their properties and toxic effects on the human body.

It is well known that PCB congeners are characterized by high bioaccumulation rates in aquatic organisms, including fishes (Chang, 2018).

Fishes are now seen as a health-promoting dietary component since they are sources of wholesome protein and provide a number of vitamins and microelements, but above all they contain polyunsaturated fatty acids (PUFA), n-6 and n-3, especially EPA and DHA.

The efficacy of n-3 highly unsaturated fatty acids, principally EPA and DHA, in the prevention or modulation of many inflammatory conditions prevalent in the developed world is well established. However, there is concern that POP contamination (e.g., dioxins, PCBs, or PBDEs) and the presence of toxic metals (e.g., Pb, As, Cd, and Hg) present potential risks to human health (Bell and Waagbø, 2008; Guo et al., 2019).

China is currently the world’s largest exporter of fishery products. Along with many other European countries, Poland imports large quantities of Chinese fishes and fish products that are purchased by a wide range of consumers because of their relatively low prices. The most common species imported to Poland are Nile tilapia (*Orechromis niloticus niloticus*), panga (*Pangasius pangasius*), Pacific cod (*Gadus macrocephalus*), pollock (*Pollachius virens*), and yellowfin sole (*Limanda aspera*). The volume of Polish imports is estimated to be approximately 260,000 tonnes of frozen fish fillets and fish products annually, which places Poland at the top of European importers (Łuszczek-Trojnar et al., 2015). Because of the scale of its fishery product exports, it is crucial to note that China was the world’s largest producer of OCPs and PCBs between 1945 and 1983. According to recent Chinese investigations, both OCPs and PCBs are still detected in the environment and in food products (Huang et al., 2019; Zeng et al., 2014).

Based on IARC/WHO (2016), PCBs are still found as posing real threats. This is evidenced by the 2016 change in the classification of dl-PCBs to group 1 compounds that are carcinogenic to humans (IARC/WHO, 2016), and the EFSA (2018) lowered the TWI value seven-fold from 14 to 2 pg-TEQ/kg b.w./week.

The aim of our study was to analyze the content of organochlorine compounds (OCPs, ndl-PCBs, dl-PCBs including mono-ortho and non-ortho congeners) in imported fish fillets and to assess the potential risk to Polish consumer health according to the new TWI values.

## Materials and methods

### Sampling

The research material was collected from batches of frozen fillets without skin in glazes of five species of fish—Nile tilapia (*Orechromis niloticus niloticus*), panga (*Pangasius pangasius*), Pacific cod (*Gadus macrocephalus*), pollock (*Pollachius virens*), and yellowfin sole (*Limanda aspera*) mainly imported from China and Vietnam obtained in 2017–2018. Individual batches of fillets (the number of batches for each fish species are in Table 1) came from different producers (the list of producers is known to the authors) and differed in the quantity of glaze (according to manufacturer declarations). The glaze is a protective layer of water added to the surface of frozen seafood. Each batch weighed about 2.5 kg. A total of 790 samples, 10 from each batch, were tested.

**Table 1:** Characteristics of the material tested

Species of fish	Amount of material tested	Country of origin	Glaze content, %	Fat content, %	Dry weight, %
Nile tilapia	18^b^ (180^c^)	China	30.71 ± 1.89^a^	1.39 ± 0.47	18.39 ± 1.71
Panga	27 (270)	Vietnam, China	27.50 ± 4.63	1.09 ± 0.73	10.86 ± 2.19
Pacific cod	13 (130)	China	27.50 ± 3.54	0.50 ± 0.06	16.79 ± 0.29
Pollock	12 (120)	China	35.00 ± 0.00	0.67 ± 0.09	8.60 ± 1.42
Yellowfin sole	9 (90)	China	25.00 ± 0.00	1.07 ± 0.17	11.25 ± 1.13

^a^Mean ± standard deviation

^b^Number of fillet batches

^c^Number of samples

### Analytical methods and instrumentation

Samples from the frozen batches of glazed fish fillets were delivered to the Department of Toxicology of the Faculty of Food Sciences and Fisheries, West Pomeranian University of Technology in Szczecin, where they were labeled and stored at − 18 °C until analysis. Prior to analysis, the fish samples were homogenized and freeze-dried for about 36 h in a LyoLab 3000-type lyophilizer (temperature − 60°C).

Determinations of the content of the compounds tested were performed on 5 g of lyophilized samples, each of which was analyzed in triplicate. The analytical procedure in Witczak and Ciereszko (2006) was applied to extract lipids containing OCPs and PCBs, and GC/MS (HP 6890/5973) was used to purify and identify compounds. For extracting the examined compounds together with lipids, a 50-cm^3^ acetone/n-hexane solution (v/v) (2.5:1) was used each time and again a 50-cm^3^ n-hexane/diethyl ether solution (v/v) (9:1). The samples were purified by adding fuming H_2_SO_4_ (7% SO_3_ in concentrated H_2_SO_4,_ w/w). The sample was concentrated to 0.1 mL under a soft nitrogen flow. Part of the samples was fortified with a known amount of single PCB congeners and pesticides to identify the examined compounds correctly and to determine recovery. Analyses were performed in three replications with the following GC/MS settings: carrier gas-helium; pressure: 0.061 Mpa (8.9 psi); flowrate: 0.8 mL min^−1^, column (HP-5MS, 5% phenyl methyl siloxane/60.0 m; ID 250 μm, 2.25 μm film thickness of the active phase) oven temperature: start from 90°C (0.5 min), increase 7°C min^−1^; 220°C (12 min), increase 6°C min^−1^; 285°C (7 min), increase 5 °C min^−1^; and 295°C (6 min) (post run). The analysis time of one sample was 54.9 min. Detector-mass spectrometer (HP5973).

### Procedure evaluation

The procedure was evaluated according to Witczak (2013) and Tomza-Marciniak et al. (2019).

To test the accuracy of the method, the certified reference material was analyzed for each batch of samples. To correctly identify the tested compounds, some samples were fortified with a known amount of each PCB congener. Mackerel oil (No 350-Community Bureau of Reference, BCR) from Promochem GmbH was used as reference material. The recovery of PCB congeners from the reference material was in the range of 79.5–91.3%.

The compound recoveries were determined with an internal standard solution of decachlorobiphenyl and 2,4,5,6-tetrachloro-m-xylene in acetone (100 μL, 80 ppb) (Pesticides Surrogate Spike Mix (4–8460, Supelco, USA). Additionally, PCB recoveries were verified using the addition of a ^13^C-labeled PCB standard solution (^13^C12-labeled PCB Mixture-A, CIL—Cambridge isotope laboratories, Inc. EC-4938) (3,30,4,40-TetraCB; 3,4,4′,5′-TetraCB; 2′,3,4,4′,5-PentaCB; 3,3′,4,4′,5-PentaCB; 3,3′,4,4′,5,5′-HexaCB; 2,2′,3,4,4′,5,5′-HeptaCB) (50 μL, 120 ppb). The average recoveries ranged from 71.8% (PCB 81) to 97.5% (PCB 180). The recoveries of compounds for which isotopically labeled standards were not available were estimated using the recoveries from samples fortified with the PCB congeners analyzed. The average recovery levels of non-ortho and mono-ortho PCB congeners were PCB 77–80.5%, PCB 126–69.8%, PCB 169–88.5%, PCB 114–74.2%; PCB 156–81.1%, PCB 157–83.5%, and PCB 81–77.7%, while the average recoveries of ndl-PCB congeners (IUPAC No PCB 28, 52, 101, 138, 153, 180) ranged from 75.2 to 89.8%. The recovery of internal standards ranged from 84.1 to 98.3%. An additional control of the identification and quantification of the analyzed compounds was based on the following standard solutions: (1) 6 PCB—Key Isomers LGC Ltd. NE 5575 (PCB IUPAC: 28, 52, 101, 118, 138, 153); (2) 12 PCB—CERTAN© NE 5570 LGC Ltd. (IUPAC No PCB 77, 81, 123, 105, 114, 126, 156, 157, 180, 169, 167, 189); and (3) Chlorinated Pesticides Mix-Supelco USA 4–9151 (αHCH, βHCH, γHCH, heptachlor, aldrin, heptachlor epoxid isomer B, dieldrin, pp’DDE, op’DDD, pp’DDT, pp’DDD, op’DDT, endrin).

The limit of detection (LOD) for each compound was determined as the concentration in the extract that produced an instrumental response to two different ions monitored with a signal to noise ratio of 3:1 for the less sensitive signal (Commission Directive, 2002/63/EC).

A blank method was included for every ten samples. The LOD for each pesticide was 0.01 ng mL^−1^, on average. The limits of quantification (LOQ) of PCB congeners were 0.03–0.1 ppb, and for organochlorine pesticides (OCPs)—as follows: heptachlor epoxide, dieldrin, pp’DDE (0.06 ppb); pp’DDD (0.05 ppb); endrin (0.03 ppb); *α*, *β*, *γ* HCH, pp’DDT (0.01 ppb); and heptachlor (0.4 ppb). The average recovery of the organochlorine pesticides ranged from 69.8 (endrin) to 98.9% (pp’DDT).

Statistical testing included determining the arithmetic means of PCBs and OCPs together with standard deviations, SD, medians, and coefficient of variation, CV, analysis of variance (Statistica 13.1 software package), and calculating Pearson’s correlation coefficient (*p* < 0.05) and Duncan’s significance test (*p* < 0.05).

### Hazard quotient

Estimations of human health risks associated with exposure to chloroorganic pesticides were assessed with the LADD (lifetime average daily dose) Eq. (1) and HQ (hazard quotient) Eq. (2) parameters (Witczak & Abdel-Gawad, 2014):

LADD (mean daily dosage during life), mg/kg·day Eq. (1)

1 $$\mathrm{LADD}=\frac{c\cdot \mathrm{ADC}}{\mathrm{BW}}$$

where *C* is the average concentration of the pesticide in fish; ADC is the average daily consumption of fish, g/day per person (in Poland 34.30 g/day) (Hryszko, 2018); and BW is the average body weight (70 kg for an adult), kg.

HQ (hazard ratio) Eq. (2)

2 $$\mathrm{HQ}=\frac{\mathrm{LADD}}{\mathrm{RfD}}$$

where RfD is the reference dose (mg·kg^−1^·day^−1^).

An HQ value exceeding 1.0 indicates that it is harmful to human health.

### Toxicity equivalency

TEQ (toxicity equivalency) reports the total toxicity of dioxin-like PCBs. To obtain TEQs, the concentration of each PCB congener in a mixture is multiplied by its toxicity equivalency factor (TEF) Eq. (3), which is established by the WHO (Commission Regulation, (EU) No 1259/2011).

3 $$\mathrm{TEQ}=\sum {\left(\left[{{C}_{\mathrm{PCB}}}_{i}\right]\cdot {\mathrm{TEF}}_{i}\right)}_{n}$$

where *C*_PCB*i*_ is the concentration of i-PCB congener.

### Statistical analysis

Statistical analysis conducted with STATISTICA 13.3 included determining the significance of differences with Duncan’s test (*p* < 0.05), and the Pearson’s correlation coefficients between the lipid and dry matter contents and the compounds analyzed. The mean contents of the compounds, standard deviations, minimum and maximum values, medians, and coefficients of variation were also determined.

## Results

### Organochlorine pesticides

The present study designated 18 compounds belonging to chloroorganic pesticides in frozen fish fillets imported from China and Vietnam. The results of all compounds are presented as arithmetic averages (in ng/g wet weight) with standard deviation, minimum, maximum, and median values, and coefficients of variation (CV) (Table 2). The lipid content (from 0.32 to 2.08%) and dry matter (from 8.08 to 21.19%) were determined in the material analyzed, which depended on the amount of glaze used the content of which accounted for between 25 and 35% of the fish products analyzed (Table 1).

**Table 2:** OCPs in fish products (ng/g w.w.)

	αHCH	βHCH	ɣHCH	δHCH	Aldrin	Dieldrin	pp'DDT	pp'DDD	pp'DDE	Endosulfan α	Endosulfan β	Endosulfan sulphate	Heptachlor	Endrin ketone	Methoxychlor
Nile tilapia															
Average	0.5069	0.2222	0.1177	0.6168	0.0201	0.1356	0.0470	0.1321	0.0284	0.0285	0.0293	0.1073	0.1374	0.1077	0.1055
SD	0.1562	0.1533	0.0621	0.1740	0.0058	0.0252	0.0568	0.0184	0.0047	0.0035	0.0277	0.0547	0.0356	0.0274	0.0135
Min	0.2654	0.0765	0.0497	0.4422	0.0114	0.0868	< LOD	0.1062	0.0229	0.0233	< LOD	0.0608	0.0944	0.0738	0.0908
Max	0.8108	0.6594	0.2539	1.1263	0.0307	0.1735	0.1292	0.1597	0.0368	0.0347	0.0700	0.2329	0.2339	0.1858	0.1350
Median	0.5373	0.1681	0.0914	0.5904	0.0206	0.1396	< LOD	0.1301	0.0281	0.0276	0.0396	0.0810	0.1309	0.1032	0.1014
CV	30.82	68.98	52.73	28.21	29.26	18.64	120.74	14.00	16.63	12.56	94.59	50.99	25.96	25.48	12.81
Panga															
Average	0.4529	0.0907	0.0559	0.6663	0.0386	0.0580	0.0502	0.0661	0.0187	0.0218	0.0256	0.0535	0.1152	0.0489	0.0890
SD	0.1635	0.0729	0.0459	0.5364	0.0938	0.0195	0.0924	0.0101	0.0034	0.0062	0.0162	0.0232	0.0438	0.0214	0.0277
Min	0.1126	0.0323	0.0224	0.1790	0.0058	0.0190	< LOD	0.0523	0.0147	0.0145	< LOD	< LOD	0.0539	< LOD	0.0590
Max	0.7243	0.2829	0.1589	2.1662	0.3898	0.0851	0.3243	0.0957	0.0284	0.0409	0.0471	0.0864	0.2070	0.0823	0.1382
Median	0.4593	0.0550	0.0324	0.5050	0.0150	0.0607	< LOD	0.0637	0.0182	0.0206	0.0319	0.0586	0.1078	0.0524	0.0765
CV	36.09	80.29	82.06	80.50	243.11	33.65	184.08	15.38	18.34	28.79	63.43	43.38	38.02	43.88	31.19
Pacific cod															
Average	0.2870	0.0385	0.0292	0.2324	0.0188	0.2986	< LOD	0.1384	0.0278	0.0251	< LOD	0.0677	0.1508	0.0699	0.0885
SD	0.0365	0.0026	0.0020	0.0632	0.0030	0.0606	< LOD	0.0238	0.0016	0.0009	< LOD	0.0076	0.0188	0.0060	0.0018
Min	0.2513	0.0359	0.0269	0.1730	0.0154	0.2173	< LOD	0.1173	0.0259	0.0242	< LOD	0.0588	0.1294	0.0612	0.0870
Max	0.3382	0.0410	0.0317	0.3165	0.0218	0.3483	< LOD	0.1706	0.0298	0.0262	< LOD	0.0743	0.1745	0.0743	0.0909
Median	0.2793	0.0385	0.0291	0.2200	0.0191	0.3145	< LOD	0.1327	0.0278	0.0250	< LOD	0.0689	0.1496	0.0722	0.0881
CV	12.74	6.79	6.88	27.21	15.99	20.29	-	17.26	5.96	3.84	-	11.24	12.53	8.69	2.13
Pollock															
Average	0.2766	0.0366	0.0259	< LOD	0.0199	0.1336	< LOD	0.0930	0.0220	0.0232	0.0385	0.0770	0.1150	0.0678	0.0907
SD	0.0232	0.0045	0.0030	< LOD	0.0012	0.0069	< LOD	0.0053	0.0032	0.0020	0.0027	0.0077	0.0221	0.0049	0.0081
Min	0.2536	0.0308	0.0217	< LOD	0.0185	0.1283	< LOD	0.0876	0.0185	0.0206	0.0357	0.0697	0.0968	0.0629	0.0823
Max	0.3055	0.0414	0.0290	< LOD	0.0216	0.1430	< LOD	0.0987	0.0253	0.0250	0.0411	0.0881	0.1453	0.0739	0.1018
Median	0.2736	0.0372	0.0264	< LOD	0.0198	0.1315	< LOD	0.0929	0.0221	0.0235	0.0387	0.0752	0.1090	0.0671	0.0893
CV	8.42	12.33	11.80	-	6.27	5.18	-	5.78	14.78	8.60	6.99	10.11	19.26	7.25	8.93
Yellowfin sole															
Average	0.2868	0.0360	0.0291	0.2053	0.0114	0.1145	< LOD	0.0948	0.0213	0.0205	< LOD	0.0740	0.0817	0.0645	0.0792
SD	0.0363	0.0075	0.0051	0.0181	0.0014	0.0262	< LOD	0.0151	0.0014	0.0006	< LOD	0.0234	0.0164	0.0067	0.0074
Min	0.2379	0.0294	0.0219	0.1812	0.0094	0.0849	< LOD	0.0723	0.0198	0.0198	< LOD	0.0489	0.0670	0.0546	0.0689
Max	0.3227	0.0463	0.0333	0.2251	0.0126	0.1394	< LOD	0.1046	0.0228	0.0212	< LOD	0.1018	0.0984	0.0699	0.0867
Median	0.2933	0.0341	0.0306	0.2074	0.0118	0.1168	< LOD	0.1011	0.0213	0.0205	< LOD	0.0727	0.0808	0.0667	0.0805
CV	12.66	21.04	17.51	8.85	12.59	22.92	-	16.02	6.83	3.38	-	31.59	20.05	10.52	9.40

*min* minimum value, *max* maximum value, *CV* coefficient of variation, *< LOD* value below the detection limit

The analysis revealed the presence of almost all pesticides tested in the fish fillets, except for endrin, endrin aldehyde, and heptachlor epoxide isomer B. According to the OCP results, the concentration of HCH was the highest (Table 2). The content of HCH isomers differed in different fish species. The highest mean concentrations of β-HCH, ɣ-HCH, and α-HCH (0.118 ± 0.016 ng/g w.w., 0.222 ± 0.049 ng/g w.w., and 0.506 ± 0.049 ng/g w.w., respectively) were noted in Nile tilapia (Table 2), and the highest concentration of δ-HCH was found in panga (0.666 ± 0.149 ng/g w.w.) (Table 2). No statistically significant differences (*p* < 0.05) were determined in the content of HCH isomers in Pacific cod, pollock, or yellowfin sole. The content of pp’DDT isomer in Nile tilapia and panga ranged between 0.047 ± 0.004 and 0.048 ± 0.012 ng/g w.w. No pp’DDT isomers were noted in Pacific cod, pollock, or yellowfin sole. (Table 2). The content of pp’DDE and pp’DDD metabolites differed in all fillets analyzed. The dominant metabolite was pp’DDD with a wet weight concentration ranging from 0.064 ± 0.007 ng/g (panga) to 0.138 ± 0.024 ng/g (Pacific cod). The highest mean concentration of the pp’DDE isomer was recorded in Nile tilapia and Pacific cod fillets (0.028 ng/g w.w.); this differed significantly (*p* < 0.05) compared with the pp’DDE content in the fillets of the other fish species. The lowest concentration of pp’DDE was recorded in panga (0.019 ± 0.001 ng/g w.w.) (Table 2).

Of the five fish species analyzed, the highest average concentration of pesticide residues by wet weight was noted in the Nile tilapia fillets, while the lowest was in the pollock fillets. Particularly noteworthy was that endosulfan sulfate dominated in relation to total endosulfan. This metabolite accounted for 65% of the total endosulfan in Nile tilapia, 53% in panga, 73% in Pacific cod, 55% in pollock, and 78% in yellowfin sole. The sulfate form is the only degradation product of endosulfan α and β that is considered very toxic. This relationship is also highly stable with a long half-life (2–6 years) depending on environmental conditions. These factors render endosulfan sulfate a more dangerous compound than the parent compound (Shah et al., 2015).

Pearson’s correlation coefficient was used (*p* < 0.05) to assess the relationship between the compounds analyzed and the dry matter (%) and lipid (%) contents of the fillets tested (Table 3).

**Table 3:** Correlation (*p* < 0.05) between the content of individual OCPs and PCBs and dry weight and lipid contents

OCPs	Pearson’s correlation coefficient, *r* (*p* < 0.05)		PCB congener		Pearson’s correlation coefficient, *r* (*p* < 0.05)	
	With dry mass	With fat content			With dry mass	With fat content
$\alpha$ HCH	0.183	0.136	ndl PCB	PCB 28	0.34	0.21
$\upbeta$ HCH	0.671	0.393		PCB 52	0.42	0.49
$\gamma$ HCH	0.637	0,315		PCB101	0.27	0.22
$\updelta$ HCH	0.033	0.026		PCB 138	0.28	0.20
pp'DDT	0.266	0.236		PCB 153	0.38	0.43
pp'DDD	0.785	0.039		PCB 180	0.21	0.37
pp'DDE	0,778	0.191	Mono-ortho PCB	PCB 105	0.29	0.18
Aldrin	0.090	0.071		PCB 114	0.07	0.10
Dieldrin	0.441	0.302		PCB118	0.03	0.29
Endrin ketone	0.718	0.197		PCB 123	0.17	0.15
Heptachlor	0.445	0.087		PCB 156	0.41	0.06
Endosulfan $\alpha$	0.635	0.297		PCB 157	0.36	0.22
Endosulfan $\upbeta$	0.144	0.383		PCB 167	0.15	0.29
Endosulfan sulphate	0.463	0.014		PCB 189	0.11	0.24
Methoxychlor	0.578	0.431	non-ortho PCB	PCB 77	0.25	0.15
				PCB 81	0.18	0.06
				PCB 126	0.32	0.23
				PCB 169	0.49	0.35

Although OCPs are accumulated in adipose tissue, this correlation (*r*_w.w._ = 0.033 – 0.785) was less pronounced because of the effects of the glaze components that comprised up to 35% of the product quantity; these included stabilizers (e.g., sodium polyphosphate [E452], pentasodium triphosphate [E452], and calcium polyphosphate [E452]), acidity regulators (e.g., citric acid [E330], sodium citrate [E331], and potassium citrate [E332]), and other additives.

Fish origin had no effect on the content of the compounds analyzed in the fish products tested.

### Polychlorinated biphenyls

**Fig. 1:**
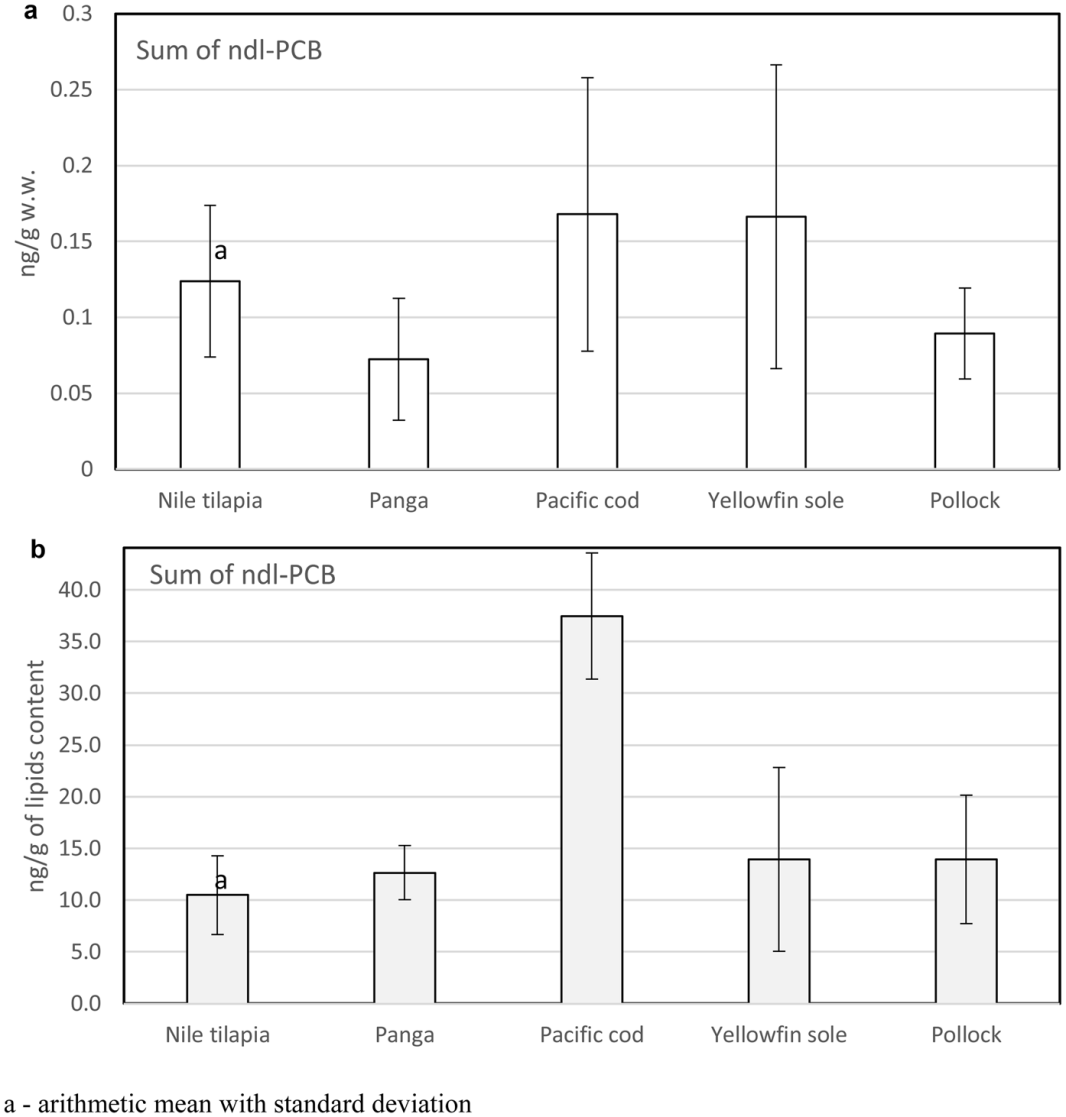
Content of ndl-PCBs in ng/g w.w. (**a**) and ng/g lipids (**b**). a—arithmetic mean with standard deviation

As most literature sources report, 90% of PCBs enter the bodied through the ingestion of contaminated foods, and the largest group of these is foods of aquatic origin (Mamun et al., 2019). Total ndl-PCBs in fish products by wet weight ranged from 0.072 ng/g to 0.168 ng/g (Fig. 1a). The highest lipid contents of total ndl-PCBs was detected in Pacific cod at up to 37.45 ng/g lipids, while the lowest levels were in yellowfin sole and pollock at 14.25 ng/g (Fig. 1b).

Dl-PCBs can be divided into mono-ortho PCBs and non-ortho PCBs. The content of ∑mono-ortho PCB ranged from 0.096 ng/g w.w. in panga to 0.160 ng/g w.w. in Pacific cod (Table 4). The content of ∑non-ortho PCBs ranged from 0.016 ng/g w.w. to 0.078 ng/g w.w. The lowest content of these compounds was detected in Nile tilapia and the highest in Pacific cod (Table 4). The content of PCBs in Pacific cod fillets differed significantly compared with that in the other fish products; however, there were no significant differences between the contents of individual PCB congeners in Nile tilapia and panga (*p* < 0.05).

**Table 4:** Content of PCB congeners in fish products (ng/g w.w.)

	ndl PCB						Mono-ortho PCB								Non-ortho PCB			
	PCB 28	PCB 52	PCB 101	PCB 138	PCB 153	PCB 180	PCB 105	PCB 114	PCB 118	PCB 123	PCB 156	PCB 157	PCB 167	PCB 189	PCB 77	PCB 81	PCB 126	PCB 169
Nile tilapia																		
Average	0.02	0.10	< LOD	0.01	< LOD	< LOD	0.01	< LOD	< LOD	< LOD	< LOD	0.01	< LOD	< LOD	0.01	0.07	0.02	0.05
SD	< LOD	0.02	< LOD	< LOD	< LOD	< LOD	< LOD	< LOD	< LOD	< LOD	< LOD	< LOD	< LOD	< LOD	< LOD	0.02	< LOD	0.01
Min	< LOD	0.10	< LOD	< LOD	< LOD	< LOD	< LOD	< LOD	< LOD	< LOD	< LOD	< LOD	< LOD	< LOD	< LOD	0.05	0.01	0.02
Max	0.03	0.16	0.01	0.01	0.01	< LOD	0.01	< LOD	0.01	0.01	0.01	0.02	0.01	< LOD	0.01	0.10	0.03	0.09
Median	0.01	0.10	< LOD	< LOD	< LOD	< LOD	0.01	< LOD	< LOD	< LOD	< LOD	0.01	< LOD	< LOD	0.01	0.07	0.03	0.05
CV	8.15	22.54	65.36	42.71	58.74	< LOD	43.32	< LOD	57.63	51.67	57.45	37.48	59.19	374.17	48.02	23.94	20.05	20.64
Panga																		
Average	0.01	0.06	< LOD	< LOD	< LOD	< LOD	0.01	< LOD	< LOD	< LOD	< LOD	< LOD	< LOD	0.02	0.01	0.04	0.02	0.04
SD	0.01	0.02	< LOD	< LOD	< LOD	< LOD	< LOD	< LOD	< LOD	< LOD	< LOD	< LOD	< LOD	0.06	< LOD	0.01	0.01	0.01
Min	< LOD	0,04	< LOD	< LOD	< LOD	< LOD	< LOD	< LOD	< LOD	< LOD	< LOD	< LOD	< LOD	< LOD	< LOD	0.02	0.00	0.01
Max	0.02	0.08	0.01	0.01	0.01	< LOD	0.02	< LOD	0.01	0.01	0.01	0.01	0.01	0.15	0.01	0.05	0.03	0.06
Median	0.01	0.07	< LOD	< LOD	< LOD	< LOD	0.01	< LOD	< LOD	< LOD	< LOD	0.01	< LOD	< LOD	0.01	0.03	0.02	0.04
CV	72.27	36.59	56.53	74.01	61.39	291.62	19.19	< LOD	76.49	82.29	109.69	61.44	82.40	313.96	49.43	29.01	36.16	17.21
Pacific cod																		
Average	0.03	0.06	0.02	0.03	0.03	< LOD	0.02	< LOD	0.02	0.03	< LOD	0.01	< LOD	< LOD	0.01	0.05	0.04	0.06
SD	0.01	0.03	< LOD	< LOD	0.01	< LOD	0.01	< LOD	< LOD	0.01	< LOD	0.00	< LOD	< LOD	< LOD	0.02	0.01	0.01
Min	6,89	13,40	4,24	5,46	6,55	0.92	3.87	< LOD	3.78	6.80	0.55	1.81	< LOD	0.63	10.63	1.93	9.45	12.60
Max	6.89	13.40	4.24	5.46	6.55	0.92	3.87	< LOD	3.78	6.80	0.55	1.81	< LOD	0.63	10.63	1.93	9.45	12.60
Median	6.89	13.40	4.24	5.46	6.55	0.92	3.87	< LOD	3.78	6.80	0.55	1.81	< LOD	0.63	10.63	1.93	9.45	12.60
CV	17,55	42,40	12,29	< LOD	33,33	< LOD	56.24	< LOD	< LOD	33.33	0.00	< LOD	< LOD	< LOD	< LOD	40.00	25.00	16.67
Pollock																		
Average	0.02	0.05	0.01	0.01	0.01	< LOD	0.01	< LOD	0.01	< LOD	< LOD	< LOD	< LOD	< LOD	< LOD	0.05	0.02	0.05
SD	< LOD	< LOD	< LOD	< LOD	< LOD	< LOD	0.01	< LOD	< LOD	< LOD	0.01	0.00	0.01	< LOD	0.01	0.01	0.01	0.02
Min	2.94	7.56	1.09	1.26	1.08	< LOD	1.54	< LOD	0.95	0.52	0.76	0.38	0.66	< LOD	0.66	7.58	0.66	8.42
Max	2.94	7.56	1.09	1.26	1.08	< LOD	1.54	< LOD	0.95	0.52	0.76	0.38	0.66	< LOD	0.66	7.58	0.66	8.42
Median	2.94	7.56	1.09	1.26	1.08	< LOD	1.54	< LOD	0.95	0.52	0.76	0.38	0.66	< LOD	0.66	7.58	0.66	8.42
CV	11.65	3.97	20.63	< LOD	< LOD	< LOD	100.00	< LOD	< LOD	< LOD	< LOD	< LOD	< LOD	< LOD	0.00	20.00	50.00	40.00
Yellowfin sole																		
Average	0.02	0.05	0.01	0.03	0.05	0.01	< LOD	< LOD	0.02	0.03	< LOD	< LOD	0.01	< LOD	< LOD	0.03	0.03	0.04
SD	< LOD	< LOD	< LOD	< LOD	< LOD	< LOD	0.01	< LOD	< LOD	< LOD	< LOD	< LOD	< LOD	< LOD	0.01	0.01	0.02	0.01
Min	1.39	4.39	0.90	2.69	4.31	0.56	0.37	< LOD	1.51	2.67	0.27	< LOD	0.78	< LOD	0.32	2.95	2.21	3.27
Max	1.39	4.39	0.90	2.69	4.31	0.56	0.37	< LOD	1.51	2.67	0.27	< LOD	0.78	< LOD	0.32	2.95	2.21	3.27
Median	1.39	4.39	0.90	2.69	4.31	0.56	0.37	< LOD	1.51	2.67	0.27	< LOD	0.78	< LOD	0.32	2.95	2.21	3.27
CV	2.52	2.45	21,94	< LOD	< LOD	< LOD	< LOD	< LOD	< LOD	< LOD	< LOD	< LOD	< LOD	< LOD	< LOD	33.33	66.67	25.00

*min* minimum value, *max* maximum value, *CV* coefficient of variation, *< LOD* value below the detection limit

Table 3 shows the correlation between the content of the compounds and the dry weight and lipid content of the fish products. Correlation coefficients were determined (*p* < 0.05) to assess the relationships between the substances analyzed and the contents of dry matter and lipids. A strong correlation was only noted between lipids (%) and PCB 52. The low correlation between the contents of individual PCBs and the lipid content could have resulted from the thick layer of glaze used to prepare the fillets or other additives.

## Discussion

Consuming fish from contaminated environments can have serious consequences for human health. To ensure consumer safety, food products are subjected to regular checks before they reach the market. However, differences in species, origin, or breeding environment can have significant impacts on the final composition of the meat (Mathew et al., 2019). Some example contents of organochlorine compounds in edible fish analyzed in different countries are shown in Table 5.

**Table 5:** Concentrations of PCBs, DDTs, HCHs, and HCB in edible fish from different regions all over the world

Sample site	ΣPCBs	ΣDDTs	ΣHCHs	HCB	References
Shantou Harbor, China	1.73	19.07	2.21	0.30	Shi et al. (2013)
Haimen Bay, China	14.91	11.00	2.63	0.15	Shi et al. (2013)
Guiyu, China	17.3	–	–	–	Xing et al. (2009)
Taizhou, China	384.5	–	–	–	Xing et al. (2010)
Dalian, China	1.11–8.04	32.82	1.17	0.38	Yang et al. (2006)
Tianjin, China	1.26–5.60	8.05	0.43	0.30	Yang et al. (2006)
Shanghai, China	0.83–11.4	19.83	0.5	0.44	Yang et al. (2006)
Pearl River Estuary, China	–	80	0.20	–	Guo et al. (2008)
Daya Bay, China	–	40	0.26	–	Guo et al. (2008)
Korea	23	8.96	0.94	0.32	Yim et al. (2005)
Australia	55	22	0.34	4.20	Kannan et al. (1995)
Spain	–	2	0.6	0.1	Lázaro et al. (1996)

### Health risk assessment of OCPs

**Fig. 2:**
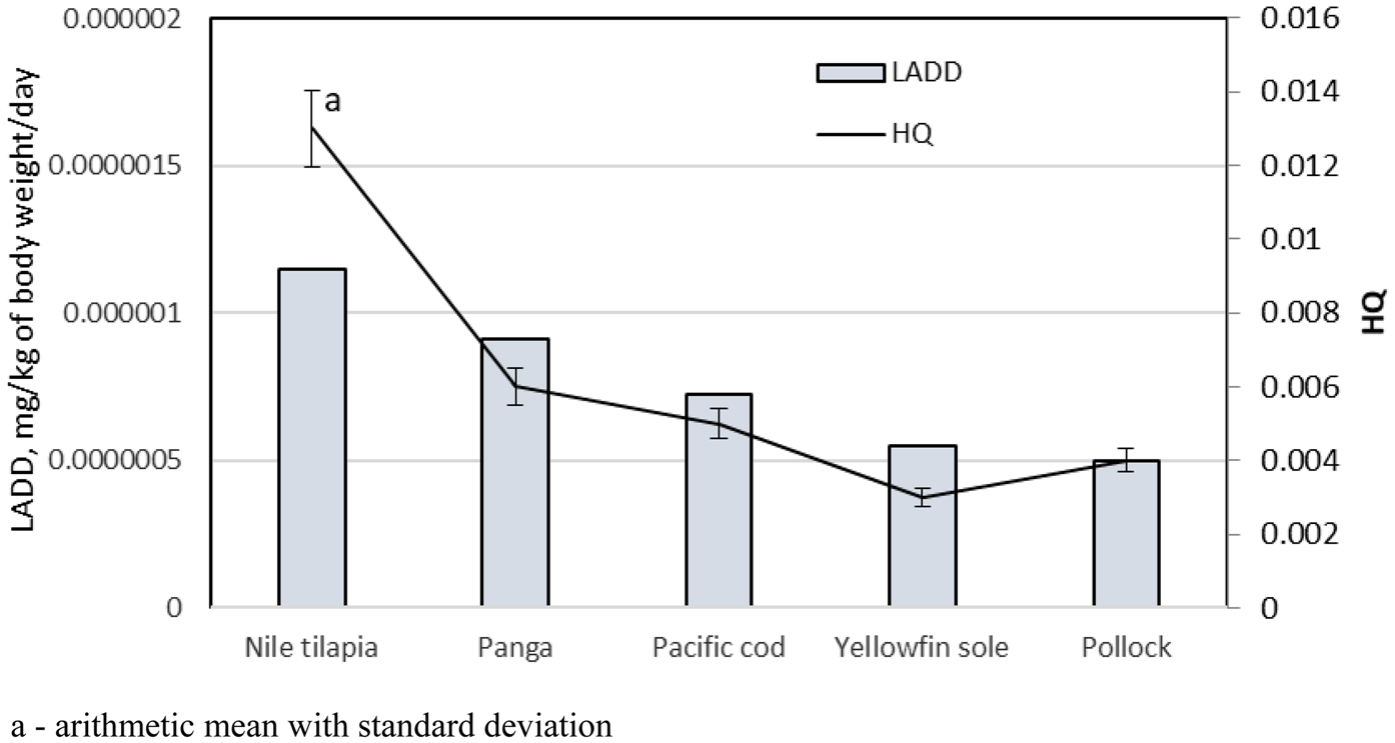
Lifetime average daily dose (LADD) and HQ (Hazard Quotient) values for fish products tested. a—arithmetic mean with standard deviation

LADD and HQ were the two parameters used in this study to determine consumer exposure to chloroorganic compounds from fish products (Fig. 2). The data obtained indicated that the risk of harmful health effects was low since the HQ hazard ratio ranged from 0.003 to 0.013. The contaminant concentrations in all samples analyzed in this experiment were also low enough so as not pose any risk to fish or consumer health. However, research from Taiwan showed samples exceeding MRL values for OCPs (Chang 2018). The positivity rate for banned OCPs in the samples from the offshore zone was 6.3% and that of imported fish was 9.4%. With some exceptions, these estimated daily intake (EDI) values were lower than 1% of the acceptable daily intake (ADI) established by the Food and Agriculture Organization (FAO) of the United Nations and World Health Organization (Chang 2018). Bioaccumulation in fish is attributed to the residues of OCPs that were used widely globally in the early 1970s. A recent study in north China indicated that the effect of OCP residuals in atmospheric particulate matter (APM) of farmland regions and aquatic environments could not be ignored since OCP residue levels in APM in certain areas contributed 41.6% to the already high health risk (Ding et al., 2015). These OCPs in APM could lead to even higher bioaccumulation in the future. Thus, the OCP concentrations in food products, especially those of fish, remain a concern.

### Health risk assessment for PCBs

Many PCBs, which are persistent and predominant in foods, are active as promoters in hepatocarcinogenesis, and lower halogenated biphenyls may be activated metabolically in vivo to genotoxic and initiating intermediates (Ludewig et al., 2008; Faroon & Ruiz, 2016). According to a report from Sweden, the estimated market basket per capita intake of total PCBs was 4.9 ng/kg body weight/day, and fish was found to be the major contributor at 64% (Törnkvist et al., 2011).

Based on EFSA (2018), in group “Fish and seafood” dl-PCB mean content was 21.0–21.6 pg WHO2005-TEQ/g whole weight. For various fish species, the mean dl-PCB level was 9.17–9.21 pg WHO2005-TEQ/g whole weight.

**Fig. 3:**
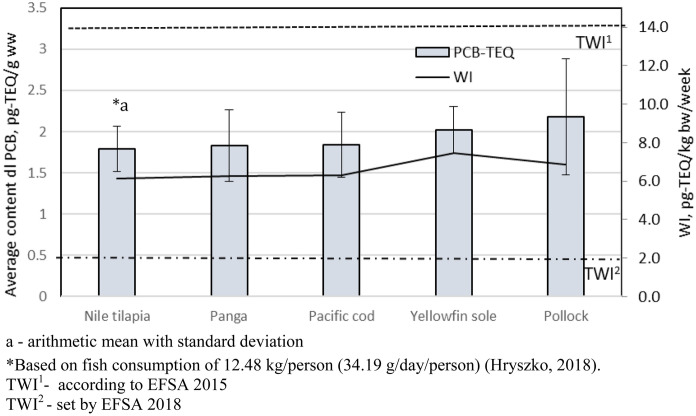
TEQ and WI assessment values in fish products. a—arithmetic mean with standard deviation. *Based on fish consumption of 12.48 kg/person (34.19 g/day/person) (Hryszko, 2018). TWI^1^—according to EFSA 2015. TWI^2^—set by EFSA 2018

Thus, it is necessary to evaluate the concentration of ∑PCB in fish products and the risk to human and animal health. The TEQ in the fish products, designated as the sum of product concentrations of individual congener TEF toxicities, ranged from 1.79 pg-TEQ/g w.w. in Nile tilapia to 2.14 pg-TEQ/g w.w. in pollock (Fig. 3).

The daily intake of PCBs from consumed fish must be estimated to assess consumer risk. According to the Institute of Agricultural and Food Economics, the annual fish consumption in 2017 in Poland was 12.48 kg/person, which was a daily intake of 34.19 g/day/person (Hryszko, 2018). The previous tolerated weekly intake (TWI) was 14 pg-TEQ/kg body weight/week or 1-4 pg-TEQ/kg body weight/day (EFSA, 2015). Based on the results of the current study, the estimated weekly intake (WI) ranged from 6.12 pg-TEQ/kg bodyweight/week in Nile tilapia to 7.45 pg-TEQ/kg bodyweight/week in pollock, which was from 43 to 53% of the TWI (Fig. 3). The new tolerated weekly intake (TWI) set by the European Food Safety Authority (EFSA Journal, 2018) means that the estimated weekly intake determined in the present study was approximately three times higher than the new TWI value (2 pg-TEQ/kg body weight/week). This is alarming since these values were obtained using only dl-PCB without dioxins. The decrease in TWI value by the European Food Safety Authority (EFSA Journal, 2018) was intended to increase the consumer safety associated with exposure to PCBs.

Assessing consumer exposure risk through food is weighty and difficult since the actual TEQ should also include toxic compounds such as polychlorinated dibenzo-*p*-dioxins (PCDDs), polychlorinated dibenzo-*p*-furans (PCDFs), and polychlorinated naphthalenes (PCNs). However, polychlorinated biphenyls constitute > 90% of the total TEQ in fishes (Alcock et al., 1998). Considering the high accumulation of PCBs, especially non- and mono-ortho congeners in aquatic organisms, as is evidenced by high TEQ toxicity rates in fishes (Ludewig et al., 2008; Lauby-Secretan et al., 2013), the results suggest that the consumer health risk is not too high. On the other hand, research in India showed that fish were highly contaminated with PCBs and posed health threats and lifetime cancer risks to consumers in the city of Hyderabad (Ahmed et al., 2016). According to an investigation by the FAO in 2013, the annual fish consumption in India of 5.8 kg per capita was similar to that in Poland (Table 6). Fish consumption has been increasing gradually globally since it contains important nutrients like EPA and DHA. Thus, even though the present study showed fish consumption to be low risk, further integrated studies are needed to gain a better understanding of PCB contamination in fish products and PCB exposure that causes health hazards. This is particularly important in the context of EFSA's six-fold reduction in the TWI parameter.

**Table 6:** Supplement. Average consumption of different fish species per capita in Poland (Hryszko, 2014, 2019)

Genre	2010	2011	2012	2013	2015	2016	2017	2018	2019^a^
	per capita in Poland (kg fresh weight)								
Nile tilapia	0.49	0.34	0.31	-	-	-	0.30		
Panga	1.47	1.21	0.89	0.81	0.55	0.49	0.50	0.29	0.30
Pollock	2.76	3.04	2.60	2.68	2.28	2.21	2.21	2.21	2.05
Pacific cod	0.42	0.65	0.83	0.85	1.22	1.11	1.04	1.00	0.85

^a^Estimated value

## Conclusion

Consuming fish from contaminated backgrounds can lead to serious consequences for human health. Species differences, origin, or breeding environment can have a significant impact on the final composition of fish meat. Fish fillets originating from imports from China and Vietnam were considered.

The results of this study indicated that although almost all OCPs were detected in all fish fillets tested, the HQ was much lower than 1.0, ranging from 0.003 to 0.013. On the other hand, although almost all PCBs were also detected in the fish tested, the estimated WI was lower than the TWI at 43–53% of TWI. These results showed that the consumption of imported fish in Poland did not pose a threat to human health. However, the estimated weekly intake values obtained in the present study were about three times higher than the TWI values set by EFSA in 2018. This raises concerns regarding consumer health risks.

The concentrations of OCPs and PCBs in fish products vary depending on fish species; these are associated with different genetic factors, metabolic pathways, and lipid contents. An important factor influencing the level of these compounds in the meat of the fish is the type of feed used to feed farmed fish.

However, processed fish products were analyzed in the current study; therefore, despite the commonly known accumulation properties of organochlorine xenobiotics in adipose tissues, low correlations between the content of these compounds and the fish product lipids were observed. We believe that the reason for this was the glaze, which comprised up to 30% of the weight of the finished product. Additionally, various additives used in the fish product glazes could also have influenced the results.
